# A Critical Analysis of the Impact of Pandemic on China’s Electricity Usage Patterns and the Global Development of Renewable Energy

**DOI:** 10.3390/ijerph19084608

**Published:** 2022-04-11

**Authors:** Muhammad Shahid Mastoi, Hafiz Mudassir Munir, Shenxian Zhuang, Mannan Hassan, Muhammad Usman, Ahmad Alahmadi, Basem Alamri

**Affiliations:** 1 School of Electrical Engineering, Southwest Jiaotong University, Chengdu 611756, China; shahidmastoi797@my.swjtu.edu.cn (M.S.M.); sxzhuang@swjtu.edu.cn (S.Z.); mannan@my.swjtu.edu.cn (M.H.); muhammadusman@my.swjtu.edu.cn (M.U.); 2 Department of Electrical Engineering, Sukkur IBA University, Sukkur 65200, Pakistan; 3 Department of Electrical Engineering, College of Engineering, Taif University, P.O. Box 11099, Taif 21944, Saudi Arabia; aziz@tu.edu.sa (A.A.); b.alamri@tu.edu.sa (B.A.)

**Keywords:** COVID-19 pandemic, electricity generation, electricity consumption, interactive analysis

## Abstract

The COVID-19 pandemic has impacted economic activity in numerous sectors due to multiple forms of disruption, including border closures, a stay-at-home policy, and social isolation; the electricity consumption trends in this region will undoubtedly improve. This article examines the impact of COVID-19 on electricity generation and consumption in China during the first two quarters (Q1–Q2) of 2020 and 2021. Furthermore, several governments’ perspectives on COVID-19’s implications for renewable energy development, notably offshore wind power and solar photovoltaics (PV), were examined. Results of this article show that COVID-19 impacts the power industry. According to the analysis, during the first two quarters of 2020, the amount of electricity generated and consumed by China decreased by 1.4 and 1.3 percent, respectively, the capacity of the power plants increased by 5.3 GW and coal consumption dropped by 3.6 g/kWh. Investments in the power generation sector increased by 51.5 billion yuan and investment in the power grid grew by 0.7 billion. Additionally, new generation capacity decreased by 378 GW during the first two quarters of 2020. During the first two quarters of 2021, electricity consumption and production grew by 13.7 and 16.2 percent, respectively. Power plants’ capacity increased by 9.5 GW, while coal consumption for power supply fell by 0.8 g/kWh. The investment in power generation projects increased by 8.9 billion, while investment in power grid projects increased by 4.7 billion. Compared to last year’s same period, 14.92 GW of new capacity was installed. Due to lockdown measures, such as studying at home or working at home, domestic power use in the first two quarters of 2020–2021 increased by 6.6 and 4.5 percent, respectively. To minimize COVID-19’s impact on renewable energy development and assist in building offshore wind power plants, economic and financial measures have been put in place to reduce the epidemic’s effect on solar PV systems.

## 1. Introduction

### 1.1. Background and Motivation

Theorists and philosophers have discussed global crises that cause outbreaks and affect the whole world since the beginning of the 21st century. In December 2019, COVID-19 was first detected in the Wuhan province of China as a respiratory disease mimicking pneumonia; the disease spread slowly throughout China and beyond; the World Health Organization (WHO) declared it a global public health emergency in January 2020 [1,2]. To prevent the virus from spreading globally in China, lockdowns, mandatory quarantines, and mask requirements were implemented. The Coronavirus (COVID-19) pandemic has had a profound and global impact on human life. As a result, every economic sector was damaged, as if a giant tsunami hit. According to the International Monetary Fund, global economic growth global growth is forecast to moderate from 5.9 percent in 2021 to 4.4 percent in 2022, reflecting forecast revisions in the two largest economies. Several revised assumptions, such as removing Build Back Better from the baseline, withdrawal of monetary accommodation earlier, and supply shortages, increased the downward revision for the United States by 1.2 percentage points. Due to pandemic-driven disruptions caused by the COVID-19 policy and prolonged financial stress among property developers, China’s credit rating has been downgraded by 0.8 percentage points. In 2023, global growth is expected to slow to 3.8 percent [3,4,5,6]. To better understand the consequences of the healthcare crisis, we need an overview of healthcare issues. As a result, we will discuss the storyline of COVID-19 around the world in the following paragraphs. The 617,283 deaths have been reported associated with COVID-19 worldwide as of April 8, 2022, including 494,587,638 confirmed cases. A total of 11,250,782,214 vaccines have been administered as of April 4, 2022. The prevalence of COVID-19 in China is 935,640 cases, with 14,048 deaths between January 3, 2020, and April 8, 2022. In addition, 3,293,262,316 doses of vaccine have been administered as of April 1, 2022. [3,4,5,6].

Most of the new cases were reported from Europe by April 2020. Meanwhile, the number of applications approved in the United States increased rapidlyBy May 2020, the United States had become one of the countries with the highest incidence of COVID-19 due to a sharp increase in new cases. In May 2020, the highest recorded deaths were in New Jersey, California, and New York [7,8,9]. However, these statistics come from government sources and differ significantly from those predicted by simulation models. By February 2021, two billion people will have carried the disease, according to a simulation model of the Rystad Energy Institute. Although there were 105 million cases officially reported (there are different statistics due to confirmed cases). According to the test model, only 5 percent of actual cases are documented in official statistics (according to the test model). There is no clear indication of how long this virus outbreak will last or what its adverse economic consequences will be. This section analyses the effects of the crisis on electricity production and consumption in the upstream part of the power sector [7,8,9].

As shown in the above facts, COVID-19 has swept the globe. Several studies, including that conducted by Faust [10], suggest that the COVID-19 pandemic may be even more severe than the 1918 Spanish flu pandemic, which killed more than 1.7 million people in 222 countries. After reaching more than 75 million confirmed cases by mid-2020, more than 1.7 million deaths had been reported to the World Health Organization by early 2020 [11]. To counteract the pandemic of COVID-19, many measures have been implemented, including quarantine, social isolation, and lockdown [12]. There has been a negative impact of COVID-19 on the power sector, agriculture, manufacturing, finance, education, health care, sports, and tourism sectors [13]. Scientists are working to minimize COVID-19’s economic and social impacts by developing more efficient methods for controlling infectious diseases. Several effective ways of controlling infectious diseases were proposed in 2020. As a result of recent developments, SARS-CoV-2 with drone technology was detected early without human intervention by 2020 [14]. Many breakthroughs have occurred in modeling and understanding COVID-19 transmission between 2020 and 2021, which are expected to help control the infection [15,16]. Even though the disease has been handled, it is still causing economic damage to many businesses and economies [17,18]. The power sector was severely affected by the disease, and this was one of the most important areas of the energy sector. In 2020 the electricity industry was not immune to the harm caused by these shocks. According to the International Energy Agency (IEA) data, electricity demand has been shocked on a large scale for the first time in seven decades and the world’s energy demand in 2020 sank by 6% compared with 2019, a drop more than seven times higher than during the financial crisis of 2009 [19].

Based on comparing the average production of 16 European countries with the average production of the previous five years, we saw a drop of 9% (25 GW) in April 2020. In this period, conventional energy production declined by 28% (24 GW), nuclear energy by 14% (11 GW), and renewable energy by 15% (15 GW) [18]. There is no doubt that energy demand is declining overall, but its effects on different types of energy and consumption habits are complex. Power and energy sectors are on their way to identifying emerging opportunities and understanding the complex impacts. Different focuses have been discussed in existing literature and studies with changes and challenges. A study published by Brosemer [20] provided a perspective on the interconnections of inequity, indigenousness, and health associated with the current energy and power crisis. Another study by Zhong [21] reviewed the implications and challenges of COVID-19 on the electricity sector. The system operators reported a significant burden due to increased uncertainty about electricity demand. The social research on energy during and after several pandemics has been examined by Fell et al. [22], who analyzed considerations, challenges, and responses during and after pandemics. According to Marstropietro et al. [23], global actions were taken to protect power consumers during the pandemic. There are many studies on specific problems in particular countries or regions, such as an impact analysis on the electricity industry in the U.S. [24], an evaluation of government interventions in South Africa [25], and an evaluation of renewable energy investments in Malaysia [26], and an evaluation of India’s electricity sector [27]. A review of the impact of containment measures on European electricity consumption is provided in [10]. In contrast, an overview is provided in [28], and an examination of the impact on energy demand in China is provided in [29,30]. Multiple perspectives were considered in studies such as the one mentioned above to understand the impact of COVID-19. More dedication is crucial in the urgent and emergent environment created by the COVID-19 outbreak. It is in the interest of the whole power/energy industry and society to provide more viewpoints.

### 1.2. Contribution and Organization

China’s power consumption patterns in upstream and downstream sectors and its market conditions are explored in this study, and the global impacts of the COVID-19 outbreaks. Several lessons are presented regarding international renewable energy development and potential business opportunities. Data for this article is based on the latest available data from the relevant agencies. Because of fast developments, the data are not accurate enough to define quantitative models. This article aims to provide a comprehensive view of electricity demand, consumption, generation, and supply in the post-COVID-19 era and to discuss new ideas for managing strategic energy in the absence of adequate data for quantitative models. Considering the prevailing circumstances of COVID-19, the study addresses the following issues:

- Explain and illustrate the global impacts of COVID-19 on renewable energy and the environment.
- For the first two quarters of 2020 and 2021, China’s electricity demand, consumption, generation, and supply are analyzed during the global spread of COVID-19.
- In 2020 and 2021, a detailed breakdown of electricity generation and consumption by quarter, according to regions and industries is provided and analyzed.
- In order to minimize the impact on the electricity sector, recommend appropriate preventative, remedial, and policy measures.

The rest of this paper is organized in the following manner. Section 2 outlines the methodology of the systematic literature review. A discussion of the effect of the COVID-19 pandemic on the sustainability of the power sector is presented in Section 3. A discussion of CO_2_ emissions is presented in Section 4. Section 5 includes the results and discussion. The electricity consumption for the first two quarters of 2020–2021 is discussed in Section 6. The comparison of China’s five-year energy development goals and UN SDG is provided in Section 7, and the conclusion is in Section 8.

## 2. Methodology

**Figure 1:**
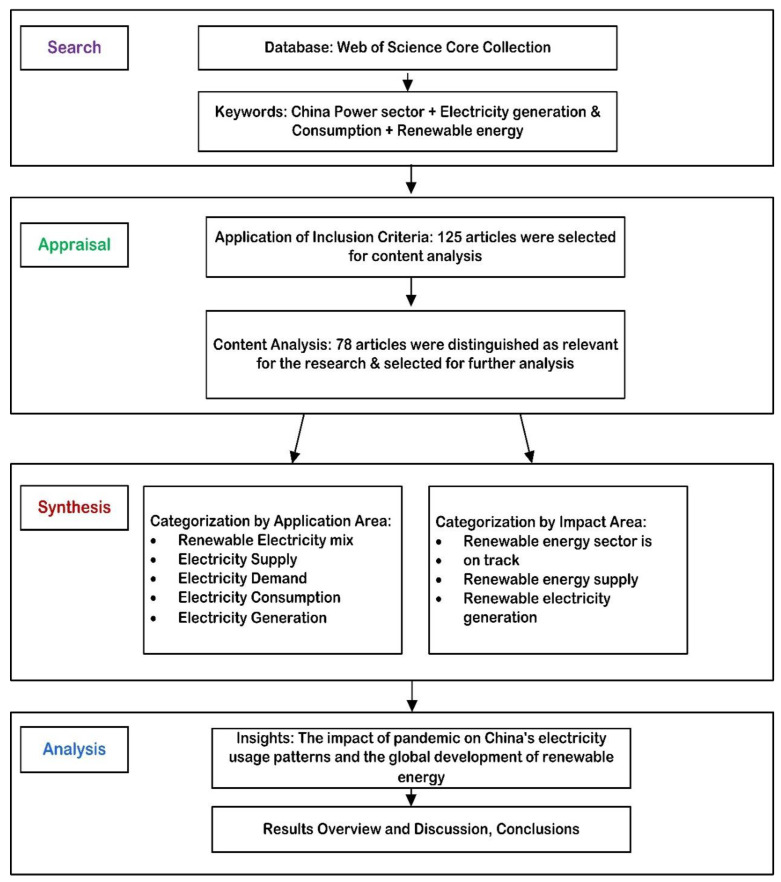
An overview of the methodology used in the study.

This study uses the official Chinese government data to analyze the trends in electricity production and consumption during the first two quarters of 2020-2021. In order to examine the COVID-19 effects on the power industry, statistics will be compared on energy production and consumption from the first two quarters of the year 2020 with those from the first two quarters of the year 2021. A database has been created in China by the China Electricity Council [31] and the Ministry of Ecology and Environment [32] that includes information on electricity production and consumption, renewable energy policies, climate change activities, and COVID-19 mitigation measures. An overview of the reviewed studies is given in Table 1, while Figure 1 shows the review’s methodological structure.

## 3. Literature Review

A detailed examination of the available studies allowed us to identify the areas of power sector sustainability where the COVID-19 pandemic had the most significant impact. The classification of studies by application and impact areas is shown in Table 2. Following are the main findings of the studies on changes in air quality, investment in renewable energy, electricity demand, electricity supply, electricity consumption, and the contribution of renewables to the energy mix.

### 3.1. Renewables

#### 3.1.1. The Renewables Sector Bucked the Trend in 2020

Renewable energy grew by 3% in 2020 as the demand for other fuels declined. Renewable energy sources generated approximately 7% more energy in 2020. Although there was reduced electricity demand, a lack of supply chain delays, long-term contracts, privileged grid access, and continuous new plant installations have all contributed to the growth of renewable energy [60].

Therefore, renewable energy’s share of global electricity generation increased from 27% in 2019 to 29% in 2020. Additionally, low oil prices reduced blended biofuel consumption, offsetting the rise in bioenergy consumption [41].

#### 3.1.2. The Renewable Energy Sector Was on Track to Set New Records in 2021

**Figure 2:**
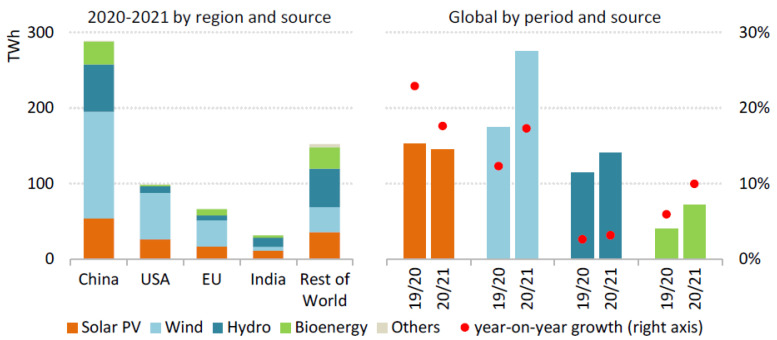
Electricity generation from renewable sources increases by technology, country, and region in 2020–2021 [61]. Source: IEA (2021) Global Energy Review 2021. All rights reserved.

By 2021, renewable electricity generation is expected to exceed 8% to 8300 TWh, the highest since the 1970s. Wind and solar PV will generate more than two-thirds of the world’s new renewable energy. Nearly half of the increase in renewable electricity worldwide will be generated by China by 2021, followed by the United States, the European Union, and India [61], as shown in Figure 2 below.

Renewable generation from wind is expected to grow by 275 TWh, or almost 17%, a substantial increase over 2020 levels. As a result of policy deadlines in China and the United States, developers finished a record amount of capacity at the end of the fourth quarter of 2020, pushing generation upswings in the first two months of 2021. The United States is expected to produce 400 TWh of wind energy in 2021 and China is expected to produce 600 TWh a year by 2030 [62]. The United States is expected to continue to expand the PV market with ongoing federal and state policy support. China is expected to remain the largest PV market. The India PV market is expecting to recover rapidly in 2021 after experiencing a significant decline because of delays caused by COVID-19, while policy incentives are driving growth in Brazil and Vietnam. As solar PV production increases by 145 trillion watt-hours, or 18%, it is expected to surpass 1000 TWh in 2021 [43].

Combined with economic recovery and the completion of large projects in China, we expected hydropower generation to increase in 2021. We expected bioenergy to gain traction in Asia due to incentives for electricity generated from waste. With increased power generation from renewable sources, 30 percent of electricity generated in 2021 will come from renewables. In combination with nuclear power, low-carbon energy sources will exceed coal generation worldwide in 2021 [40,63].

#### 3.1.3. Supply of Renewable Energy in the First Two Quarters of 2021 as Compared to the First Two Quarters of 2020

Renewable energy consumption increased by 1.5% during the first two quarters of 2021 compared to the first two quarters of 2020. At the same time, renewable electricity generation increased by about 3%. The number of solar PV and wind projects completed by 2020 will be over 100 GW and 60 GW, respectively. Moreover, in the first half of (Q1–Q2) 2020, the United States and Europe had excellent wind availability. Furthermore, renewable energy is resilient to lower electricity demand since its lower operating costs and priority regulations allow it to be dispatched before other electricity sources [42,64].

Between Q1 and Q2 of 2021, renewable energy accounted for almost 28% of global electricity production, compared to 26% in the same quarter of 2020. Renewable energy has largely replaced coal and gas, providing more than 60% of global electricity. Solar PV and wind power generated 9% of the China electricity during the first and second quarters of 2020, compared with 8% in the first and second quarters of 2020 [65,66,67]. From Q1 to Q2 of 2020, variable renewable energy contributed significantly to electric power generation. Since weather conditions were favorable, several renewable energy projects were completed in 2020, and electricity demand did not grow as rapidly; variable renewable energy shares remained similar or higher even before the lockdown measures were implemented. The electricity demand dropped as soon as lockdown measures were implemented, while wind and solar PV levels remained steady. As a result, the share of variable renewable energy increased. Variable renewable electricity caused lockdowns in parts of the US, Belgium, Germany, Italy, and Hungary [65]. Since 22 March 2020, strict social distancing measures in Germany have consistently exceeded the percentage of renewable energy in the same period of 2020. Renewable energy has taken an increasingly greater share in electricity systems in the last few months because solar PV penetration rises significantly in the summer months when most markets experience higher levels.

#### 3.1.4. The Share of Renewables in the Electricity Generation Mix Grew Rapidly during the Lockdown Period

In China, during confinement, the amount of coal-fired power was drastically reduced due to a decrease in electricity demand. In the second half of March 2020, coal’s share recovered slightly with the progressive release of lockdown measures, while renewables remained a large part of the mix. Hydroelectricity generation from new capacity and heavy rains contributed to the increase in renewable energy in China during June and July. The coal and renewable generation trends changed with the availability of hydropower in the autumn. With seasonal constraints and stronger demand for electricity, coal production picked up again during November because of lower hydroelectricity generation [44].

### 3.2. Electricity

#### 3.2.1. The Electricity Demand in 2020

During the first half of 2020, commercial and industrial activities were restricted as lockdowns resulted in a decline in electricity demand of around 1%. Some lockdown periods saw a decrease of 20–30% in demand. When weather variations are stripped out, China’s demand in February decreased by more than 10% compared to the same period in 2019. As the world’s second-largest energy consumer after China, the United States saw its electricity consumption decline almost identically in May as stay-at-home orders soared. Weekly demand declined by more than 15% in Germany, France, and the United Kingdom from March to April, while Spanish and Italian demand dropped by over 25%. Indian demand declined by more than 20% from mid-March to the end of April. The number of people infected with COVID-19 in Japan and Korea decreased by around 8% in May, whereas it was a very common outbreak in Europe and the United States. While advanced economies improved in the second half of 2020, they remained below levels seen in 2019. China and India recorded year-on-year growth rates of more than 8% and 6%, respectively, in the last quarter of 2020, which was indicative of the high growth rates in emerging markets and developing regions [46].

#### 3.2.2. The Electricity Demand in 2021

Electricity demand was expected to rise by 4.5% in 2021 as the global economy recovered and emerging economies such as China grew rapidly.

**Figure 3:**
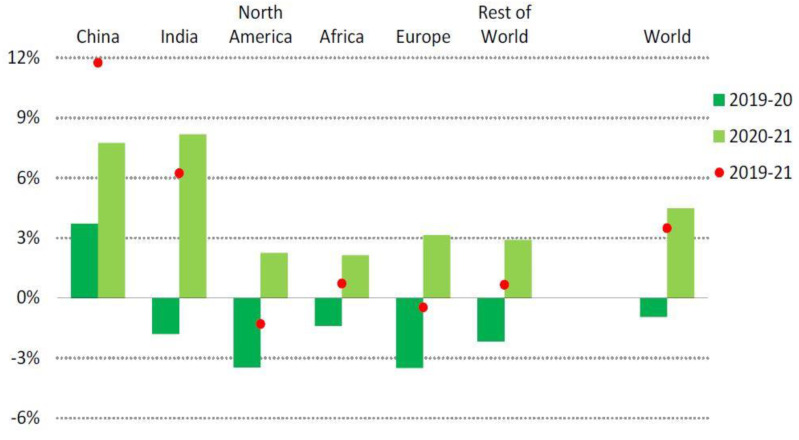
Changes in electricity demand by region between 2020 and 2021 [47]. Source: IEA (2021) Global Energy Review 2021. All rights reserved.

Advanced economies should gradually lift restrictions between spring and autumn following vaccination campaigns against COVID-19. Within 1% of current levels, the 2.5% expected growth in demand should be sufficient to keep it in line with 2019. In the first half of 2021, economists predicted that the U.S. demand for heating oil would grow by around 2%. Due to colder weather and economic stimulus, this happened. According to [47], this increase would push demand to 1.6% of its levels in 2019. Despite an increase of almost 3% in 2021, there will no longer be enough of an increase to offset declines of 4% to 6% in 2020 for the largest consumers in Europe—Germany, France, Italy, and Spain. Similar situations are expected in Japan, where demand is only predicted to increase by 1% from 2020, short of reversing the 4% decline in 2020. The second half of 2020 is predicted to continue to see growth in demand for goods and services in emerging and developing economies. With China and India’s economic recovery expected to be strong, this trend will be accelerated. China’s GDP was projected to grow by 9% in 2021 and India’s GDP by 12%, which would result in an 8% increase in electricity demand. China’s 2021 demand was projected to increase by almost 12% over 2019,. is on top of 2020 growth. Strong growth was expected in Southeast Asian countries, with total demand expected to grow by 3% over 2019 levels by 2021, as shown in Figure 3.

During the COVID-19 pandemic in 2021, China’s industrial and commercial electricity consumption recovered strongly. Growth is expected to return to pre-pandemic levels within all demand sectors during 2021–2024, but slower than before. In contrast, the demand for electricity for road transport is expected to continue to grow dramatically through 2024. However, it is starting from a relatively low base as electrification accelerates to meet China’s goal to end the sale of new internal combustion engines by 2035. Chinese electricity is still largely coal-based despite diversifying the generation mix. Coal generated 64% of all power in 2021, followed by hydropower with 16%, wind at 7%, and nuclear at 5%. The share of coal in the mix is expected to decline to 59% by 2024. During 2022–2024, renewable energies are expected to meet most of the additional energy demand (over 70%), while coal will meet 25%. To meet the growing electricity demand, China continues to increase its generation capacity. Onshore wind and solar PV continue to be deployed rapidly, despite government subsidies being phased out. By 2024, both technologies will achieve a capacity of over 930 GW, up from close to 530 GW in 2020. Besides deploying these variable renewable energy sources, China announced plans to add more than 30 GW of new capacity for non-hydro energy storage by 2025, up from 3.3 GW in 2021.

**Figure 4:**
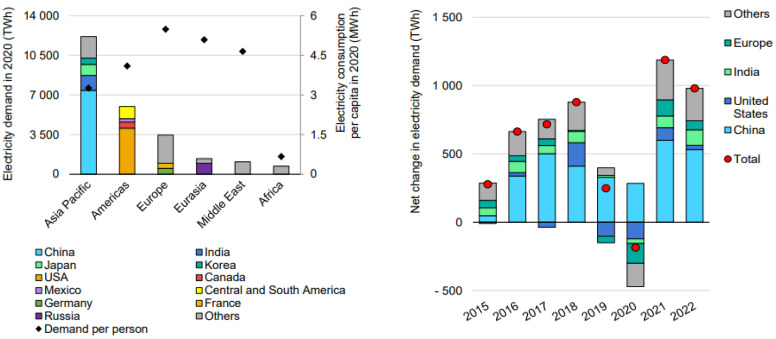
Changes in electricity demand by region in 2020–2022 [57]. Source: IEA (2021) Electricity Market Report July 2021. All rights reserved.

According to [57], 2020–2021 electricity demand declined by about 1%; in 2022, demand is expected to grow by about 4.5%, resulting in average growth of 5.1%, Growth before COVID-19 was similar to the average growth from three to five years earlier. As a result, it expects most demand growth to take place in the Asia Pacific. Over the past two decades, China has contributed most to developing electricity demand. China’s contribution to global electricity demand has increased from 10% in 2000, to 20% in 2010, to above 30% in 2020. Over 50% of the worldwide demand increase happened in China between 2000 and today. As a result, the country’s per capita consumption increased more than five-fold and is comparable with Europe’s. Even though Chinese electricity demand grew only by 4% in 2020, it was still lower than the average growth rate for the three years prior (6.6%). The consumption growth in 2022 is predicted to be 6.5% due to strong demand growth in the latter part of 2020 and the beginning of 2021. The annual electricity demand fell by 2.4% in India in 2020, the third-largest electricity consumer after China and the United States. The lowest demand was recorded in March when it fell by 23% year-over-year. Electricity consumption per capita in India is just over 1 MWh per year, well below the regional average of 3.3 MWh. It shows potential for future growth. After a strong increase in consumption in the first quarter of 2021, demand fell in April due to a surge in COVID-19 cases. In the second half of the year, assuming demand grows at the first quarter’s pace, it could rise by more than 8% in 2022, and the United States could have recovered its electricity demand in 2021. However, it was about 0.5% below before the 2019 pandemic, following the trend over the past few years. It expects a slight growth of less than 1% for 2022. Demand declined in every European country in 2020 by at least 4%, especially in Italy, Spain, and the United Kingdom, where drops of at least 6% were recorded. Despite the cold temperatures early in the year, the demand was expected to increase by 4% in 2021 after the vaccine programs were implemented, and the commercial sector reopens by 2% in 2022. Russian electricity demand grew significantly earlier this year 2022. The country is Eurasia’s largest consumer of energy and the fourth-biggest in the world. In the first third of last year 2021, annual demand was 2.8% higher than in 2020, falling by 3%. Demand is expected to rise by about 1% in 2022, returning to recent patterns, as shown in Figure 4.

### 3.3. Electricity Supply Worldwide

#### 3.3.1. The Electricity Supply in 2020

**Figure 5:**
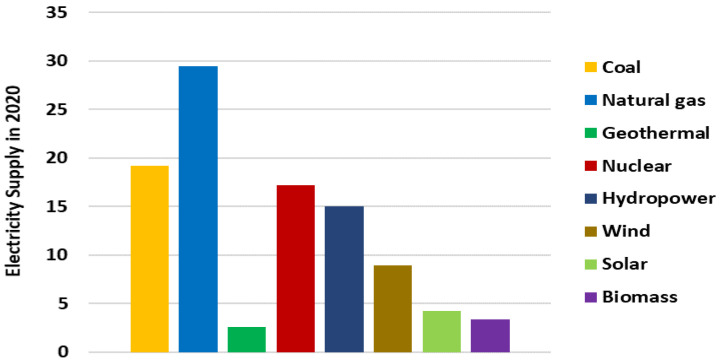
Changes in electricity generation in the year 2020.

As a result of falling, electricity demand combined with the growth of renewable energy—led by wind power and solar PV-fossil fuel and nuclear power plants—struggled a great deal in 2020. Demand for nonrenewable energy sources decreased by 3% in 2020. Electricity demand for coal in 2020 was reduced by 440 TWh, the most out of all the sources. The coal generation dropped by 4.4%, making it the greatest drop in absolute terms and the biggest drop in relative terms in the past 50 years, as shown in Figure 5.

Almost half of the global decline can be attributed to low gas prices alone in the United States, and 23% of the decline can be directly attributed to the European Union—a decline primarily offset by increases in renewable energy generation. The power generated by gas-fired plants declined by 1.6% less than coal-fired plants in 2020. A competitive price environment contributed to the lower gas prices, particularly during the middle of the year. Even though gas-fired generation increased in the United States in 2020 by 2%, coal-fired generation declined by 20%, or 210 TWh. Since 2012, the world’s oil production has declined continuously by 4.4% [38].

#### 3.3.2. The Electricity Supply in 2021

**Figure 6:**
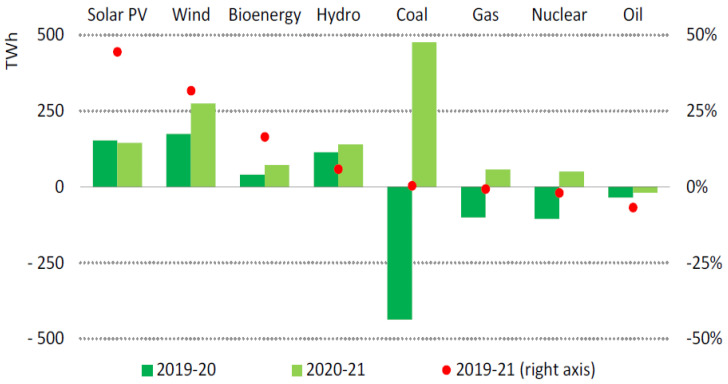
Changes in electricity generation between 2020 and 2021 [53]. Source: IEA (2021) Global Energy Review 2021. All rights reserved.

As a result of recent developments, renewable energy-based electricity generation grew for the 20th consecutive year in 2021. Renewable energy sources were expected to provide more than half of the extra electricity in 2021. Nuclear power was expected to grow by around 2%, so coal and gas plants would continue to generate the rest of the electricity demand. Electricity generated from fossil fuels was largely provided by coal-fired power plants, whose output was expected increase by 480 TWh. Despite higher gas prices, there was not much improvement in the natural gas industry (+1 According to the U.S. Department of Energy, coal-fired electricity generation declined by around 20% in 2020. In some regions, the coal-to-gas switching economy is unwinding and was expected to reverse half of these losses in 2021. The US gas-fired generation was forecast to decrease by more than 80 TWh by 2021, [68] as shown in Figure 6.

As the world’s largest coal-fired electricity producer, China was expected to generate substantially more electricity in 2021 than the rest of the world. Although China accounted for almost half of the global renewable energy supply in 2021, about half was expected to come from fossil fuels. As a result, coal consumption in China was predicted to rise by 330 TWh (or 7%) from 2019. It was predicted that India would have the second-largest absolute growth rate after China as of 2021, with 70 percent of the additional electricity demand met by thermal generation, mostly from coal [53].

### 3.4. Electricity Demand in the First Two Quarters of 2021 as Compared to the First Two Quarters of 2020

Despite lockdown measures in most countries for less than a month, the electricity demand fell 2.5% in the first and second quarters of 2021 [52]. With the first containment measures in mid-January2021, China achieved the largest reduction in demand of 6.5% during Q1–Q2 2021. In March 2021, restrictions were introduced gradually in other parts of the world, but they had a much lower impact. In the first half of 2021, Europe, Japan, Korea, and the United States saw a drop of 2.5% to 4% in electricity demand compared with the first half of 2020. This is due to COVID-19 and milder temperatures in January and February [58].

When full lockdowns were conducted, electricity demand dropped by 20% or more. Partial lockdowns had a smaller impact. In the United Kingdom, the Southwest of the U.S., France, India, Italy, and Spain, daily electricity consumption dropped by at least 15% after full lockdowns were implemented [54]. In economies where strict measures were adopted and countries with more effective services, these impacts were most noticeable. For example, Italy experienced a 25% decrease in electricity consumption due to these measures. There was only a marginal impact on electricity demand when the partial lockdown was implemented and continued in Japan. During the containment phase in Europe and the United States, a maximum percentage of 10% was permitted [68].

### 3.5. Electricity Supply in the First Two Quarters of 2021 Compared to the First Two Quarters of 2020

With depressed electricity demand because of lockdown measures, renewable energy has gained a significant share of electricity generation. In Q1–Q2 2021, there was a 2.6% decline in global electricity generation compared to Q1–Q2 2020. Renewable-based generation increased by 3% in 2020, especially wind power, which experienced a double-digit percentage increase, and new solar PV projects significantly increased generation [55]. The amount of renewable energy in the electricity supply reached 28% in the first half of 2021, up from 26% in the first half of 2020.

Most electricity sources declined in the first two quarters of 2021, except renewables, which were largely unaffected by electricity demand. In some regions, fewer reactors were operational due to decreased demand. This led to a decrease of 3% in nuclear power production. Low-carbon energy production increased overall, allowing fossil fuel-produced electricity to be reduced by approximately 3% [48]. Low prices for natural gas boosted the growth of gas-fired generation worldwide by 4%. The switch from coal to gas first became an opportunity in certain markets due to fuel costs. There were many obstacles to coal power generation in the first two quarters of 2021, with output falling by 8% compared to the same period in 2020 [49].

Between Q1 and Q2 of 2021, all regions with lockdown measures implemented toward low-carbon energy sources transformed their electricity supply. China is the world’s largest coal-power generator; it had the biggest decline in coal-based power generation, about 100 TWh per year, contributing to the global decline. China experienced pronounced reductions in February and March, during which substantial lockdowns took place. Due to a demand reduction, renewables have gained a share in power generation in the European Union in recent weeks. Because of this, coal and gas have been pushed out of the energy mix [33,34,35,50,51,52,69,70]. Following the initiation of lockdown measures in the U.S., coal-powered generation decreased more rapidly. During the same period, gas-fired generation decreased slightly, and renewable generation increased. Accordingly, U.S. coal-fired generation decreased by one-third in Q1–Q2 2021 over Q1–Q2 2020 due to lower demand, cheap gas, and an increase of 20% in wind and solar PV production. The coal power generation in India and its share in the national power mix declined sharply after nationwide measures with immediate effect were implemented from Q1 to Q2 2020 to Q1 to Q2 2021, bringing coal and renewables shares in electricity generation closer than ever before [36,37,39].

## 4. CO_2_ Emissions

**Figure 7:**
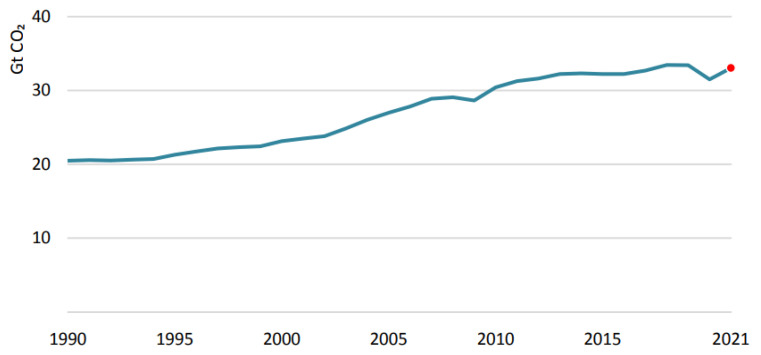
Energy-related CO_2_ emissions in 2020–2021 [73]. Source: IEA (2021) Global energy-related CO_2_ emissions, 1990–2021. All rights reserved.

CO_2_ emissions dropped by 5.8% in 2020, or almost 2 Gt CO_2_ -- the greatest decrease in recent history and five times more significant than the decline following the global financial crisis in 2009. Since oil and coal suffered more than other energy sources from the pandemic in 2020, CO_2_ emissions decreased more than energy demand. But renewable energy sources have become more significant. Although global energy-related CO_2_ emissions declined slightly in 2020, the annual average concentration of CO_2_ in the atmosphere in 2020 averaged 412.5 parts per million -- about 50% higher than when the industrial revolution began. By 2021, global emissions of CO_2_ were projected to rebound by 4.8% as coal, oil, and natural gas prices rebound with the economy, as illustrates in Figure 7. CO_2_ emissions are expected to increase by over 1.5 billion metric tons, the largest increase since recovery from the global financial crisis. It would reduce global emissions in 2021 by only 400 metric tons from the 2019 peak, representing a 1.2% reduction. Energy demand and emissions increased in China in 2020. CO_2_ emissions increased by around 500 Mt. Compared to 2019, Chinese CO_2_ emissions increased by almost 600 Mt in 2021. All fossil fuels were responsible for a higher amount of CO_2_ emissions in China in 2021. Coal was expected to dominate, contributing 70% of the increase in electricity due to higher coal use in the electric sector. Between 2019 and 2021, coal-fired power plant output increased nearly 7% in China despite rapid growth in renewable energy [71,72]. 

### 4.1. China’s Emissions Trading System (ETS)

China has been operating its national emissions trading system (ETS) since 2021 when it was launched in 2017. Initially covering the power sector, which contributes over 40% of China’s energy-related CO_2_ emissions, the ETS will then be extended to cover other energy-intensive industries. Chinese climate goals, such as achieving carbon neutrality by 2060 and reaching a peak in CO_2_ emissions by 2030, can be met with the help of a national ETS. Using the Chinese ETS can reduce emissions from power generation and assist the power sector in its transformation. China’s power system is discussed in detail at national and provincial levels, based on comprehensive national and regional scenarios for 2020–2035.

### 4.2. China’s ETS and Its Role in Decarbonizing the Power Sector

China’s ETS is intended to reduce CO_2_ emissions from power plants, increase thermal plant efficiency, and ensure peak emissions are reached. It is designed to work with four types of power plants: unconventional coal-fired units; conventional coal-fired units below 300 MW; conventional coal-fired units above 300 MW; and gas-fired power plants. Based on Table 3, benchmark designs for 2020 and 2021 would reduce average CO_2_ emissions intensity from 2015 by approximately 3.5%. Meanwhile, ultra-supercritical units’ average CO_2_ emissions intensity would be lower than their benchmark designs. 

Energy units with carbon capture and storage (CCS) that use coal or gas are subject to the same benchmarks. 85% of CO_2_ is captured and stored by an average carbon capture and storage unit (CCS). As more high-efficiency coal-fired units are deployed, CO_2_ emissions will gradually decrease. The use of low-efficiency or older units will generally end when they have reached the end of their useful life (estimated at 30 years) or when they are no longer economically viable. If the benchmarks remain unchanged, there will be surpluses of CO_2_ allowances, which will lead to a lack of incentives to reduce CO_2_ emissions from coal and gas-fired units. In order to ensure that the national ETS functions correctly, the ETS Scenario assumes benchmarks will be gradually tightened over time. It is planned that benchmarks for coal-fired and coal + CCS power plants will be gradually lowered in the first few years to ease the transition to the new system and reduce shock to market participants. A tightening rate increase is scheduled for 2025 to support peaking the power sector’s CO_2_ emissions before 2030 [74].

Several possible impacts of the ETS on the Chinese power sector have been analyzed using three scenarios:

- The No-Carbon-Pricing Scenario is used to evaluate the role of the ETS. A no-carbon-pricing scenario assumes no specific policies to control CO_2_ emissions, including the ETS or command-and-control approaches such as emissions caps or consumption standards; however, it assumes that power can be economically dispatched from 2025 and that wind and solar PV capacity can be deployed at the required levels.
- The ETS Scenario is the main scenario used to assess China’s role in its power sector. Additionally, this scenario utilizes the assumptions from the No-Carbon-Pricing Scenario, but in 2020, a national ETS will be imposed for electricity production with free allowances. In addition, it assumes that benchmarks for coal-fired technologies will be lowered in the long run. Under China’s current plan for ETS allowance allocation, gas-fired units with surplus allowances will not be required to purchase additional allowances.
- The Intensity Target Case in the No-Carbon-Pricing Scenario simulates the impact of mandatory energy consumption standards on reducing emissions. The intensity target case follows the same emission reduction trajectory as the ETS scenario.

## 5. Results Overview and Discussion

### 5.1. The Consumption of Electricity during the First Two Quarters of 2020

Between January and June, the electricity consumption by the entire society increased due to a rise in consumption in the primary industry and the residential sector. The increase in electricity consumption year on year exceeded the national average in 18 provinces. Data on energy consumption indicates the manufacturing and industrial sectors have continued to decline, according to references [45,59]. Table 4 shows that each energy-intensive industry grew in its electricity consumption from January–June 2020.

### 5.2. Electricity Generation between January and June 2020

**Figure 8:**
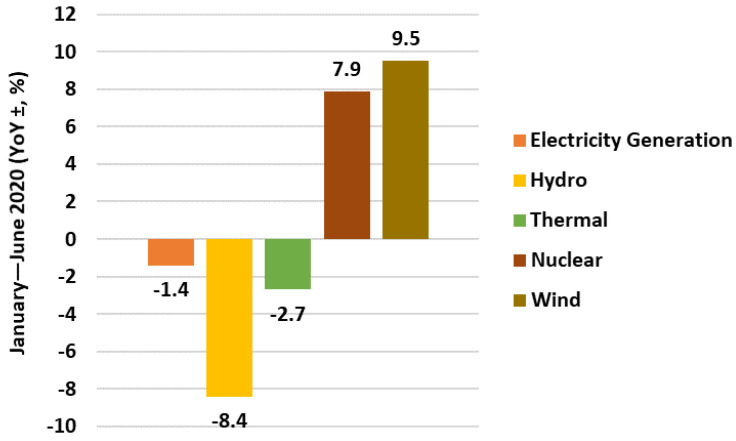
China’s electricity generation from January to June 2020.

In the period from January to June, electric power generation nationwide was 3364.7 TWh, representing a decrease of 1.4% year on year, as shown in Figure 8.

The total amount of electricity generated between January and June was 494.5 TWh, down 8.4% year-on-year; the total amount of thermal electricity generated from January to June was 2451.9 TWh, decreased by 2.7% year-on-year; the total amount of nuclear electricity generated was 189.2 TWh, grew by 7.9% year-on-year; and the total amount of wind electricity generated was 229.1 TWh, increased by 9.5% year-on-year.

### 5.3. The Consumption of Electricity across Society

**Figure 9:**
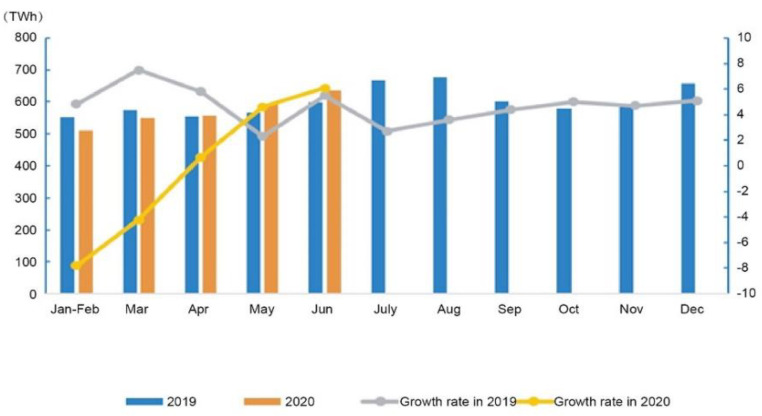
Electricity consumption and its growth rate by month in 2019 and 2020 [37]. Source: CEC (2020) Statistics of Electricity Consumption. All rights reserved.

Electricity consumption at a national level was 3354.7 TWh between January and June, a decline of 1.3% from the previous year. Figure 9 shows that nationwide electricity consumption in June reached 635.0 TWh, a 6.1% increase over the previous year.

**Figure 10:**
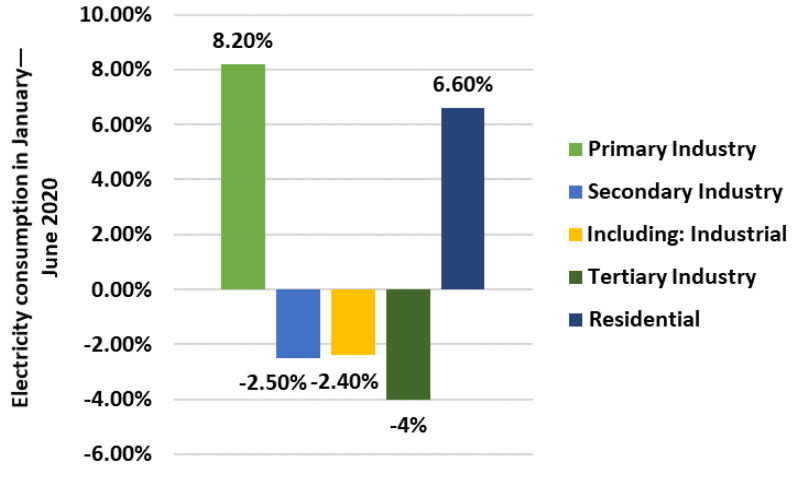
Sector-wise electricity consumption in January–June 2020.

Sector-wise, electricity consumption for the primary industry was 37.3 TWh from January to June, an increase of 8.2% year-on-year; that for the secondary industry was 2251.0 TWh, a decrease of 2.5% year-on-year; for the tertiary industry, it was 533.3 TWh, a decrease of 4.0%; and that for residential use was 533.1 TWh, an increase of 6.6% year-on-year as illustrated in Figure 10.

### 5.4. Electricity Consumption by Regions

**Figure 11:**
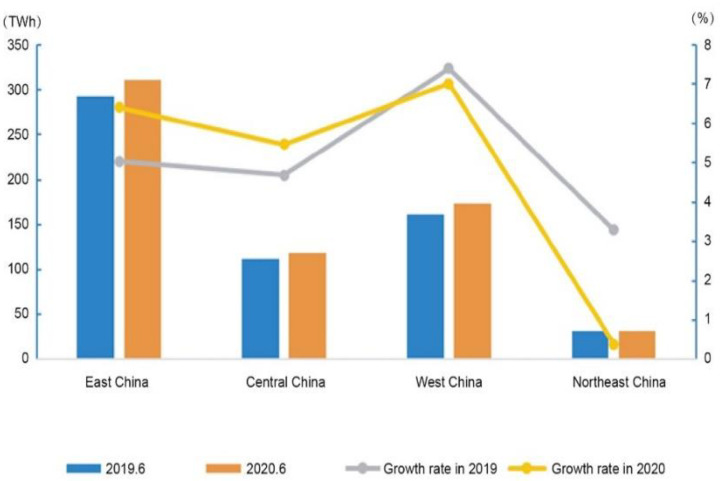
The growth rate of electricity consumption by region in the years 2019–2020 [45]. Source: CEC (2020) Statistics of Electricity Consumption. All rights reserved.

In the eastern, central, western, and northeast regions of China, respectively, the electricity consumption from January to June was 1548.5 TWh, 627.8 TWh, 975.5 TWh, and 202.9 TWh, with year-on-year growth rates of −3.1%, −3.0%, 2.9% and −0.5%. For details, see Figure 11.

**Figure 12:**
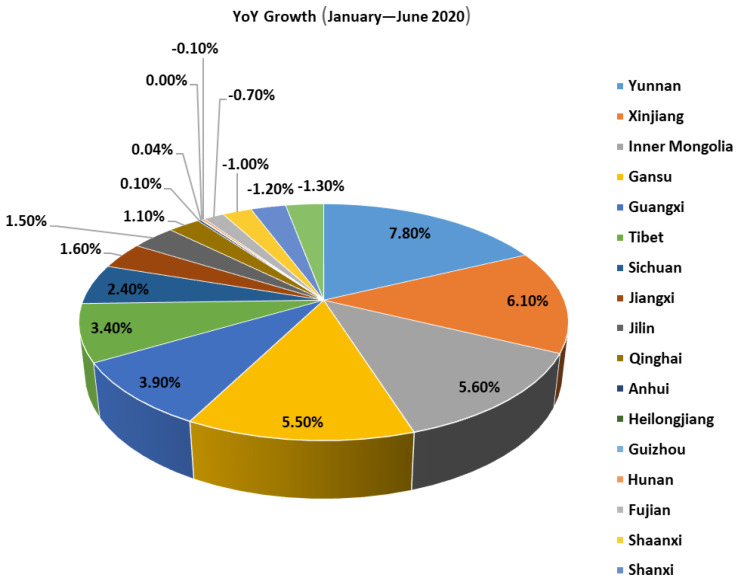
Electricity consumption by region in 2020 in terms of YoY growth.

In 18 provinces recorded year-on-year increases in electricity consumption above the national average from January to June: Yunnan (7.8%), Xinjiang (6.1%), Inner Mongolia (5.6%), Gansu (5.5%), Guangxi (3.9%), Tibet (3.4%), Sichuan (2.4%), Jiangxi (1.6%), Jilin (1.5%), Qinghai (1.1%), Anhui (0.1%), Heilongjiang (0.04%), Guizhou (0.0%), Hunan (−0.1%), Fujian (−0.7%), Shaanxi (−1.0%), Shanxi (−1.2%) and Chongqing (−1.3%) See Figure 12 and Table 5 for details.

### 5.5. Electricity Consumption in the Manufacturing Industry

According to the Energy Information Administration, the National Industrial Electricity Consumption for January to June was 2211.6 TWh, a decline of 2.4% compared to the same period in the previous year. The growth rate declined by 5.3 percentage points. Electricity consumed by industry accounts for 65.9% of total consumption. In June, the national industrial electricity consumption was 433.4 TWh, up 4.2% year-on-year. Compared to the same period last year, the growth rate decreased by 0.6 percentage points, representing 68.2% of total electricity consumption.

**Figure 13:**
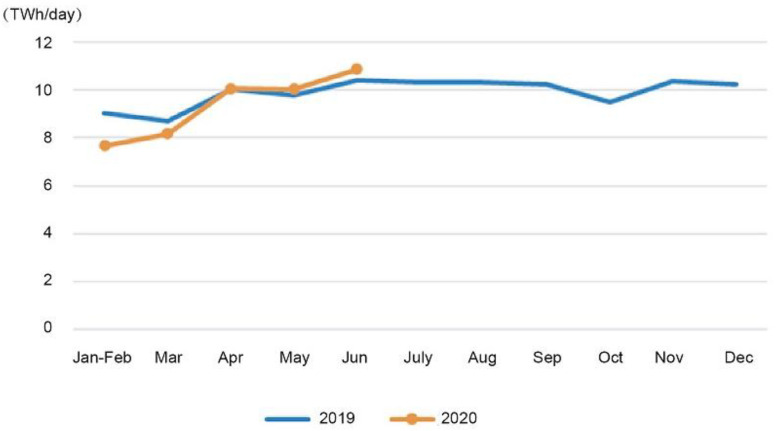
Manufacturing’s average daily electricity consumption by month from 2019 to 2020 [37]. Source: CEC (2020) Statistics of Electricity Consumption. All rights reserved.

During the same period of the previous year, the manufacturing sector consumed 1666.0 TWh of electricity, a decrease of 3.0%. Over that period, growth dropped by 6.4 points. The total power consumption in the four energy-intensive industries in the same period last year was 947.2 TWh, a 1.0% decline and a growth rate of 4.4 percentage points. The high-tech and equipment manufacturing sector’s electricity consumption dropped by 4.4 percent, and the growth rate dropped by 7.9 points from 330.7 TWh during the same period last year. The consumption of electricity in the consumer goods manufacturing industry declined by 9.4% on a year-on-year basis to 210.8 TWh, and the growth rate declined by 11.5 percentage points from last year’s same period. As shown in Figure 13, other manufacturing sectors consumed 177.3 TWh of electricity during the same period of last year, a reduction of 2.5% from the previous year and a growth rate of 7.5%.

### 5.6. Electricity Consumption in Energy-Intensive Industries

Among the chemical industry,213.5 TWh of electricity was consumed from January through June, a decrease of 3.2% over the comparable period last year. This industry also experienced a decline of 4.5 percent in growth. The building materials industry consumed 162.9 TWh of electricity last year, down 4.4% over last year. The growth rate slowed by 10.5%. The amount of electricity consumed in iron and steel smelting and refining was 275.4 TWh, a decrease of 0.4% year-on-year and a 6.3 percent decrease from last year. Electricity consumption in the non-ferrous metal smelting and refining industry was 295.4 TWh, a rate of growth of 0.9 percentage points over the same period last year and an increase of 2.1% from last year.

## 6. The Consumption of Electricity during the First Two Quarters of 2021

Power consumption increased rapidly in society between January and June 2021. Power consumption in the three industries grew faster than society’s average power consumption level. In 15 provinces, electricity consumption grew faster than the national average. The manufacturing sector consumed more electricity daily than ever before. All four energy-intensive industries increased their electricity consumption by double digits [75,76,77]. A full analysis of the first two-quarters of China’s power sector can be found in Table 6. This table shows all the types of electricity produced from renewable and nonrenewable sources, along with power consumption in different industrial zones and residential areas.

### 6.1. Electricity Generation in January and January–June 2021

**Figure 14:**
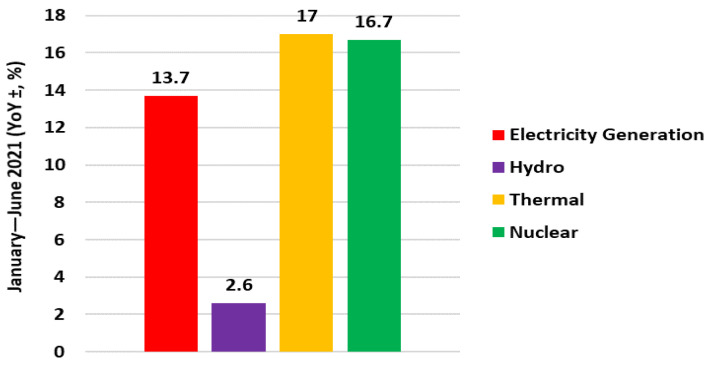
Electricity generation in January–June 2021.

The electricity generated from January to June was 3871.7 terawatt-hours, increasing 13.7% year-on-year. As shown in Figure 14, hydroelectricity generated 604.7 TWh of electricity from January to June, an increase of 2.6% year on year; thermal electricity generated 2948.2 TWh of electricity, an increase of 17.0% year on year; and nuclear electricity generated 317.1 TWh of electricity, an increase of 16.7% year on year. An analysis of different electricity generation sources during the first two quarters of 2021 is below.

### 6.2. Overall Electricity Consumption in 2021

**Figure 15:**
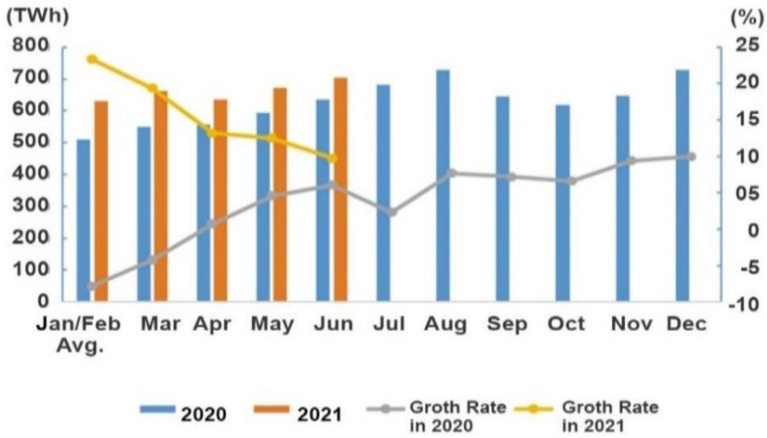
The industry’s electricity consumption from January to June in 2021 [75]. Source: CEC (2021) Statistics of Electricity Consumption. All rights reserved.

The overall electricity consumption in 2021 was 3933.9 TWh, an increase of 16.2% YoY, with a monthly consumption in June was 703.3 TWh, an increase of 9.8% YoY, as shown in Figure 15. The chart shows China’s electricity consumption month-by-month for the last year.

**Figure 16:**
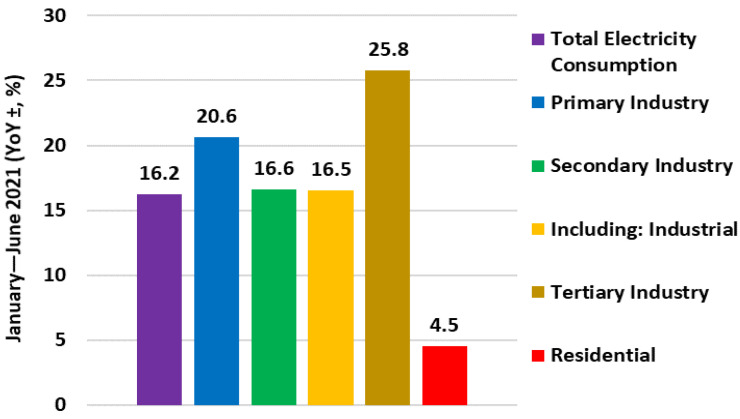
Sector-wise electricity consumption in January–June 2021.

Figure 16 shows the annual electricity consumption for the different sectors from January through June; the primary industry consumed 45.1 TWh, a 20.6% increase; the secondary industry consumed 2661.0 TWh, a 16.6% increase; the tertiary industry consumed 671.0 TWh, a 25.8% increase; and residential consumers consumed 556.8 TWh, a 4.5% increase. In the first two quarters of 2021, tertiary industry consumes the most electricity, and residential areas consume the least.

### 6.3. Electricity Consumption by Region

From January to June, 1858.1 TWh of electricity was consumed in the Eastern, Central, Western, and Northeastern parts of China, 733.9 TWh, 1119.5 TWh, and 222.4 TWh, respectively. Growth rates in the four regions were 17.7%, 16.9%, 14.8%, and 9.6%, respectively.

**Figure 17:**
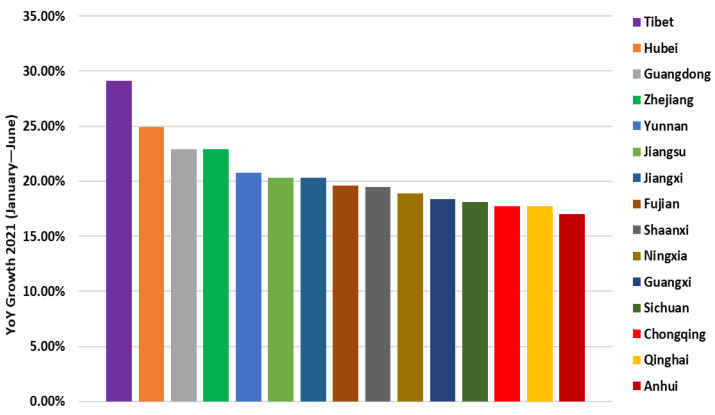
The growth of electricity consumption by region in 2021.

15 provinces recorded higher growth rates in electricity consumption than the national average, as shown in Figure 17 and Table 7: Tibet (29.1%), Hubei (24.9%), Guangdong (22.9%), Zhejiang (22.9%), Yunnan (20.8%), Jiangsu (20.3%), Jiangxi (20.3%), Fujian (19.6%), Shaanxi (19.5%), Ningxia (18.9%), Guangxi (18.4%), Sichuan (18.1%), Chongqing (17.7%), Qinghai (17.7%) and Anhui (17.0%). The region of China with the highest energy consumption is Tibet, and the region with the lowest is Anhui [75].

### 6.4. Electricity Consumption in Industrial and Manufacturing Sector 

From January through June, the total industrial energy consumption was 2612.7 TWh, constituting 66.4%. The increase was 16.5% on an annual basis, or 18.9 percentage points. In June, industrial electricity consumption hit 474.7 TWh, increasing by 8.5% year on year. This represents a 4.3 percentage point increase compared to last year, making up 67.5% of the nation’s electricity consumption.

**Figure 18:**
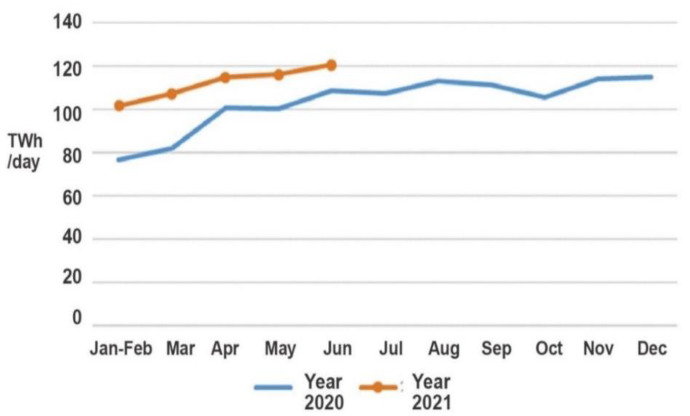
Electricity Consumption by the industrial and manufacturing sectors in 2020–2021 [75]. Source: CEC (2021) Statistics of Electricity Consumption. All rights reserved.

The manufacturing industry consumed a total of 1998,0 TWh of electricity during the first six months of 2021. This represents an increase of 18.4% over the same period of the previous year and an increase of 21.4 percentage points over the precursor period. As a result, the four energy-intensive industries consumed 1096.4 TWh, an increase of 13.7% compared to last year and an increase of 14.8 percentage points compared to the same period last year. During the same time period, the high-tech and equipment manufacturing industry consumed 421.6 TWh, a 27.3% increase over the year before. A 31.8 percentage point increase was recorded over the same period last year. A total of 262.3 TWh of electricity were used by the manufacturing industry, increasing 22.2% over last year and 31.6 percentage points over the year before. As illustrated in Figure 18, the electricity consumption of other manufacturing industries increased 22.3% in a single year from 217.3 TWh to 217.3 TWh.

### 6.5. Electricity Consumption in Energy-Intensive Industries

**Figure 19:**
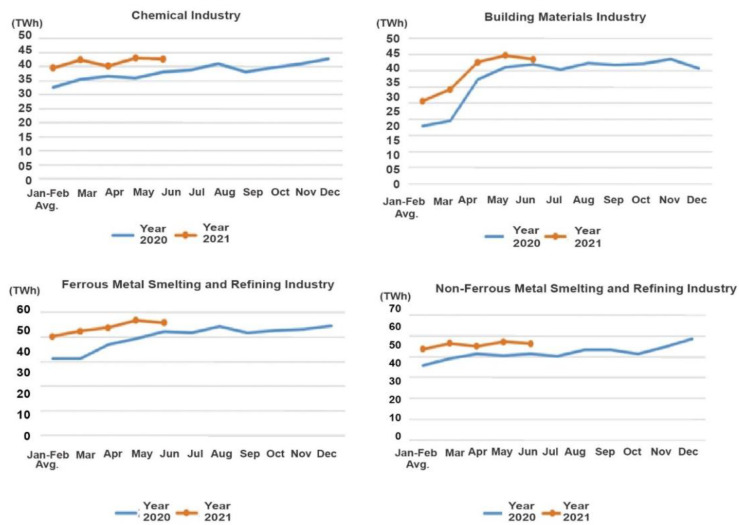
Monthly electricity consumption of key industries in years 2020–2021 [75]. Source: CEC (2021) Statistics of Electricity Consumption. All rights reserved.

Over the period from January to June, the chemical industry utilized 247.5 TWh of electricity. This increased 12.0% from the same period last year and a 15.2 percent increase. During the same period last year, the building materials industry consumed 196.7 TWh of electricity, an increase of 19.8% from the previous year, or a 24.2 percentage point increase. Smelting and refining of iron and steel resulted in the consumption of 319.9 TWh of electricity, an increase of 16.1% compared to last year, representing an increase of 16.5 percentage points. Figure 19 shows the increase in electricity consumption for the non-ferrous metal smelting and refining industry, which grew by 9.6%, or 7.5 percentage points, from the same period last year. According to the literature, industrial electricity consumption in 2021 was higher than in 2020.

## 7. China Energy Development Goals Compared with UN SDGs

In December 2016, the National Energy Administration adopted the 13th Five Year Plan for Renewable Energy Development (2016-2020) to advance energy deployment until 2020. Targets were set in accordance with the 13th Five Year Plan on National Economic and Social Development and the respective five-year plans for each renewable energy technology [78].

- As of 2020, non-fossil energy contributed 15% of total primary energy consumption; by 2030, this was up to 20%.
- As of 2020, renewable energy has reached 680 GW of installed capacity.
- Wind power capacity is expected to reach 210 GW by 2020.
- Encourage the development of offshore wind and ocean energy.
- Promote technological innovations in renewable energy.
- Enhance the development of China’s renewable energy industry and reduce its dependence on foreign companies.
- Fix the curtailment issue related to renewable energy.

The United Nations General Assembly adopted several Sustainable Development Goals (SDGs) in September 2015 as part of the 2030 Agenda for Sustainable Development. The new Agenda focuses on achieving sustainable development for every person by "leaving no one behind.". Goals for affordable and clean energy are as follows:

- In order to ensure universal access to clean, affordable, reliable, and modern energy by 2030.
- Globally, renewable energy will contribute considerably to the energy mix by 2030.
- Improvements in energy efficiency are expected to double by 2030.
- To promote the development of clean energy technology and research, such as renewable energy, energy efficiency, and enhancements in fossil-fuel technology, and to encourage the investment in clean energy infrastructure, international cooperation should be strengthened.
- In addition to expanding and upgrading infrastructure, the goal is to provide efficient and sustainable energy services to all developing countries by 2030, particularly the Least Developed Countries, Small Island Developing States, and Landlocked Developing Countries.

## 8. Conclusions

**Figure 20:**
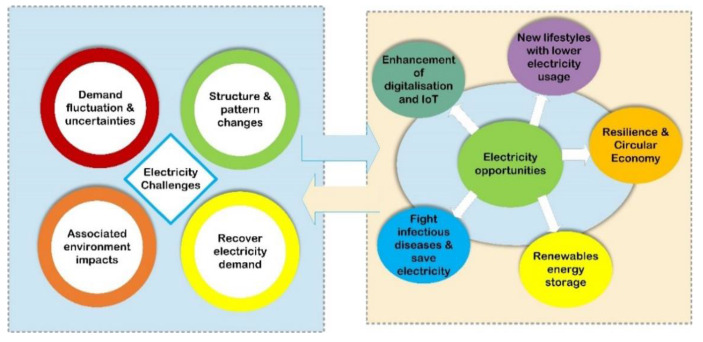
Based on COVID-19, the power sector has several challenges and opportunities.

A pandemic caused by COVID-19 has become a profound challenge for humanity worldwide. The global economy relies heavily on trade and movements, and these movements have adversely affected modern society. Energy, the lifeblood of modern society, also has a negative impact on the environment. Furthermore, there is also an issue regarding the environment’s health and medical, business, and commercial concerns. These challenges are expected to continue growing and impact the energy sector even more, as shown in Figure 20. An analysis of the effects of the COVID-19 pandemic on electricity consumption and demand reveals the following point-by-point observations:

- In the first two quarters of 2020–2021, we observed a structural shift in a number of electricity demand factors, including short-term compared to long-term expectations, residential and non-residential consumption patterns, consumption philosophy, consumed products, and electricity intensities across regions.
- There are heterogeneous characteristics in the electricity recovery in different regions. When the pandemic was well-controlled in China, the electricity consumption returned to normal about three months after the lockdown measures were relaxed. While similar three-month durations of lockdown have been observed in the U.S. and Japan, pandemics are repeating or worsening in these two countries. Despite the increase in daily COVID-19 cases, India is nearly back to its normal electricity consumption level. The European Union is on its way to restoring electricity demand. Policy, sociological factors, and geographic factors all contribute to the time it takes for electricity to recover.
- Due to outbreaks in the first two quarters of 2020, China’s electricity production and consumption decreased by 1.4 percent and 1.3 percent. Power plant generation capacity increased by 5.3 GW, coal consumption decreased by 3.6 g/kWh, investment in power generation projects increased by RMB 51.5 billion, investment in power grid projects increased by RMB 0.7 billion, and newly installed generation capacity decreased by 378 GW. In the first two quarters of 2021, the electricity generation and electricity consumption were up 13.7 and 16.2 percent, power plant capacity increased by 9.5 GW, and coal consumption decreased by 0.8 g/kWh. There was an increase in investment in power generation projects of RMB 8.9 billion, while investment in power grid projects increased by RMB 4.7 billion. Compared to last year’s same period, 14.92 GW of new capacity was installed. Demand for energy in the residential sector increased, while demand in the commercial and industrial sectors decreased. Peak demand times have shifted in response to changes in consumption profiles. Environmental quality has improved.

### 8.1. Policy Recommendations

Following are policy recommendations regarding the current demand for electricity, the availability of generation fuel, and the sustainable development of power enterprises:

1. Ensure a balance between supply and demand during the summer.
  - This year, the power supply and demand situation is more difficult, resulting in more areas experiencing power shortages this summer than the previous year.
  - Optimize regional resource allocation by coordinating various generating units and taking advantage of their peak power generation capabilities.
  - The management of energy demand must be improved. Resources should be allocated according to the market.
  - Coordination of network sources, strengthening the grid, and reducing bottlenecks on low-voltage lines are necessary improvements.
2. Increase low-carbon power mix transformation.
  - Plans for the development of the different sectors within the power industry according to a systemic approach.
  - Promote a carbon peak for coal-fired generating capacity.
  - Ensure that a large share of renewable energy is integrated.
3. The market-oriented mechanisms and policy system to ensure the low-carbon transition of the power sector should be enhanced and improved.
  - Improve the power pricing mechanism under the power market.
  - Enhance coordination between medium- and long-term markets.
  - Carbon markets should be developed more quickly

### 8.2. Future Impacts of Pandemics on Power Sectors

- Limited time to decarbonize: The EU, U.S., and Japan have all taken 50 to 70 years to achieve carbon neutrality, whereas China has just 10 years left before it reaches the carbon peak and 30 years left until it achieves carbon neutrality.
- All sectors must reduce emissions, including those hard to decarbonize, such as steel, non-ferrous metals, petrochemicals, building materials, construction, and transportation.
- There is a dramatic increase in electricity consumption. The additional annual electricity consumption is estimated to reach 380 TWh from 2021 to 2030, equal to the total electricity consumption of the 10th largest country in the world.
- Load peaks during peak times will continue to be a challenge; technological innovations need to be made in renewable energy, ultra-high voltage, grid management, and the development of new storage systems to ensure stability.
- In 2025, total electricity consumption will be 9500 TWh, and in 2035, it will be 12,600 TWh, and in 2025 and 2035, per capita consumption will be 6640 kWh. In the next 15 years, the Compound Annual Growth Rate (CAGR) will be 3.6%.
- By 2025, 2030, and 2035, the generating capacity will be 3 TW, 3.9 TW, and 5 TW. Non-fossil fuel generating capacity will make up 52%, 60%, and 67% of total electricity generation.
- As part of future policies, solar, wind, and hydropower will be utilized in southwestern China. However, nuclear energy will become more important.

## Figures and Tables

**Table 1:** A general description of the reviewed studies.

Impact Areas	Application Areas	Methods Used	Case Studies Locations	Years of Publications
Investments in renewables	Renewable energy mix	ARDL approach (Experimental based)	Romania, Chile, Argentine, Brazil	2017
Carbon emissions/air pollution	Electricity demand	The life cycle assessment (Theoretical based)	China, India, South Korea, Taiwan	2018
Electricity Consumption	Electricity Supply	Linear regression model (Experimental based)	The United States, Australia, Canada	2019
Electricity supply	Electricity consumption	General equilibrium model (Experimental based)	Brazil, Chile, Argentine	2020
Electricity demand	Economic uncertainty	Machine learning approaches (Experimental based)	Turkey, Serbia, Bangladesh, Kuwait, Ethiopia	2021

**Table 2:** Study categorization by application and impact areas.

Impact	Renewable Energy Electricity Mix	Electricity Demand	Electricity Supply and Consumption
Carbon emissions/air pollution	[18,24,33,34,35,36]	[16,34,37]	[38,39]
Investments in renewables	[8,14,15,40]	[8,41,42]	[19,25,43,44,45]
Consumption	[28,30,38]	[11,13]	[2,10,21,46,47,48,49,50,51,52]
Electricity Demand	[53,54,55]	[12,56]	[6,27,57,58,59]

**Table 3:** CO_2_ emissions benchmark design for 2020–2021.

Category Benchmarks	Type of Technology	Benchmark CO_2_ Emissions for Electricity Generation (g/kWh)
Unconventional coal-fired power plants	Fluidized circulation bed (CFB)	989
The capacity of a conventional coal fired unit is less than 300 MW	Intensive pressure Subcritical power ≤ 300 MW Supercritical power ≤ 300 MW	907
A conventional coal-fired power plant that produces more than 300MW	Subcritical power > 300 MW Supercritical power > 300 MW Ultra-supercritical Coal + CCS	829
A gas-fired unit	Gas + CCS	376

**Table 4:** China’s power sector statistics for the first two-quarters of January to June 2020 [45]. Source: CEC (2020) Statistics of China Power Industry. All rights reserved.

Indicator	Unit	Total Values	January–June, 2020 (YoY %, ±, pp)
Electricity Generation	TWh	3364.7	−1.4
Hydropower	TWh	494.5	−8.4
Thermal power	TWh	2451.9	–2.7
Nuclear power	TWh	189.2	7.9
Wind power	TWh	229.1	9.5
Total Electricity Consumption	TWh	3354.7	−1.3
Primary Industry	TWh	37.3	8.2
Secondary Industry	TWh	2251.0	−2.5
Including: Industrial	TWh	2211.6	−2.4
Tertiary Industry	TWh	533.3	−4.0
Residential	TWh	533.1	6.6
Generating Capacity of Power Plants over 6000 kW	GW	1936.22	5.3
Hydropower	GW	316.14	2.7
Thermal power	GW	1196.06	3.8
Nuclear power	GW	48.77	6.2
Wind power	GW	216.59	12.4
Average Coal Consumption of Power Supply	g/kWh	302.6	−3.6
Line loss	%	4.54	−0.4
Heat Supply	TJ	2,682,210	4.2
Coal Consumption for Heat Supply	Mt	158.57	3.6
Electricity Supply	TWh	2876.5	−2.5
Electricity Sales	TWh	2745.8	−2.1
Power Equipment Average Utilization Hour	Hour	1727	−107
Hydropower	Hour	1528	−145
Thermal power	Hour	1947	−119
Nuclear power	Hour	3519	90
Wind power	Hour	1123	−10
Auxiliary Power Ratio	%	4.6	−0.08
Hydropower	%	0.3	0.01
Thermal power	%	5.8	−0.1
Power Generation Projects Investment	Billion RMB	51.5	51.5
Hydropower	Billion RMB	25.3	25.3
Thermal power	Billion RMB	−31.9	−31.9
Nuclear power	Billion RMB	−1.5	−1.5
Wind power	Billion RMB	152.2	152.2
Power Grid Projects Investment	Billion RMB	0.7	0.7
Newly Installed Generation Capacity	GW	−378	−378
Hydropower	GW	230	230
Thermal power	GW	−62	−62
Nuclear power	GW	−1255	−125
Wind power	GW	−277	−277
Newly Installed Substation Equipment Capacity of 220 kv and above	GVA	−2344	−2344
Newly Added Transmission Line Length of 220 kV and above	km	−1726	−1726

Unit: 10^12^ Terawatt-hour (TWh). YoY: Year on year.

**Table 5:** Based on the YoY growth in electricity consumption from January to June 2020.

Regions	YoY Growth (January–June 2020)
Yunnan	7.8%
Xinjiang	6.1%
Inner Mongolia	5.6%
Gansu	5.5%
Guangxi	3.9%
Tibet	3.4%
Sichuan	2.4%
Jiangxi	1.6%
Jilin	1.5%
Qinghai	1.1%
Anhui	0.1%
Heilongjiang	0.04%
Guizhou	0.0%
Hunan	−0.1%
Fujian	−0.7%
Shaanxi	−1.0%
Shanxi	−1.2%
Chongqing	−1.3%

**Table 6:** Statistic on China’s power sector for the first two-quarters of January to June 2021 [75]. Source: CEC (2021) Statistics of China Power Industry. All rights reserved.

Indicator	Unit	Total Values	January–June 2021 (YoY %)	±	pp
Electricity Generation	TWh	3871.7	13.7		
Hydropower	TWh	604.7	2.6		
Thermal power	TWh	2948.2	17.0		
Nuclear power	TWh	317.1	16.7		
Total Electricity Consumption	TWh	3933.9	16.2		
Primary Industry	TWh	45.1	20.6		
Secondary Industry	TWh	2661.0	16.6		
Including Industrial	TWh	2612.7	16.5		
Tertiary Industry	TWh	671.0	25.8		
Residential	TWh	556.8	4.5		
Installed Generation Capacity (Year to Date)	GW	2256.60	9.5		
Hydropower	GW	377.85	4.7		
Thermal power	GW	1266.58	4.1		
Nuclear power	GW	52.16	6.9		
Wind power	GW	291.92	34.4		
Solar power	GW	267.61	34.4		
Average Coal Consumption of Power Supply	g/kWh	301.4		−0.8	
Line loss	%	4.4			−0.2
Heat Supply	TJ	2,974,620	6.6		
Coal Consumption for Heat Supply	Mt	178.60	9.1		
Electricity Supply	TWh	341.55	17.7		
Electricity Sales	TWh	326.61	17.9		
Power Equipment Average Utilization Hour	Hour	1853		119	
Hydropower	Hour	1496		−33	
Thermal power	Hour	2186		231	
Nuclear power	Hour	3805		286	
Wind power	Hour	1212		88	
Solar power	Hour	660		−3	
Auxiliary Power Ratio	%	4.5			−0.1
Hydropower	%	0.3			0.01
Thermal power	%	5.7			−0.1
Power Generation Projects Investment	Billion RMB	189.3		8.9	
Hydro power	Billion RMB	47.5		19.1	
Thermal power	Billion RMB	20.2		10.3	
Nuclear power	Billion RMB	22.6		44.3	
Wind power	Billion RMB	82.6		−3.2	
Solar power	Billion RMB	16.5		12.2	
Power Grid Projects Investment	Billion RMB	173.4		4.7	
Newly Installed Generation Capacity	GW	51.87			14.92
Hydro power	GW	8.12			4.00
Thermal power	GW	17.57			1.25
Nuclear power	GW	2.27			2.27
Wind power	GW	10.84			4.52
Solar power	GW	13.01			2.86
Newly Installed Substation Equipment Capacity (Ac 220 kv and above)	GVA	137.33			28.39
Newly Added Transmission Line Length (>220 kV)	km	19,882			5317

Unit: 10^12^ Terawatt-hour (TWh). YoY: Year on year.

**Table 7:** Regional growth in electricity consumption from January to June 2021.

Regions	YoY Growth (January–June 2021)
Tibet	29.1%
Hubei	24.9%
Guangdong	22.9%
Zhejiang	22.9%
Yunnan	20.8%
Jiangsu	20.3%
Jiangxi	20.3%
Fujian	19.6%
Shaanxi	19.5%
Ningxia	18.9%
Guangxi	18.4%
Sichuan	18.1%
Chongqing	17.7%
Qinghai	17.7%
Anhui	17.0%
